# Supplementary material for: A PRISMA systematic review of adolescent gender dysphoria literature: 1) Epidemiology
Source: PLOS Glob Public Health. 2022 Mar 9;2(3):e0000245. doi: 10.1371/journal.pgph.0000245 (PMC10021877; doi:10.1371/journal.pgph.0000245)
Supplement: Attachment — Submitted filename: Response to reviewers.docx [file pgph.0000245.s002.docx]

**THE SAHLGRENSKA ACADEMY**

**Institute of Neuroscience and Physiology**

**Gillberg Neuropsychiatry Centre**

**Kungsgatan 12**

**411 19 Göteborg**

Dear Runsen Chen

We very much appreciate the timely and constructive comments from reviewers on our manuscript. We have addressed each of their points in turn in the table below. All documents have been uploaded in the appropriate format. We hope this will allow our paper to be published in the near future. Please note, we have now submitted the second paper in this review series (focused on mental health comorbidity) to your journal (ref: PGPH-D-21-00929), taking into account points made by reviewers here.

|  | **Comment** | **Response** |
| --- | --- | --- |
| 1.1 | The searched timeframe in different databases are varied (“we searched Ovid Medline 1946 – October week 4 2020, Embase 1947–present (updated daily), CINAHL 1983–2020, and PsycInfo 1914–2020.), I wonder what could be the reasons the authors chose different start and end time for them. Did they affect the final article selection? | As is usual practice in a systematic review, all searches were conducted on the same day. The start date of each database is a product of that database and its coverage, and not a matter of our choice. Similarly, the end dates available on the day of search will vary slightly due to the way databases are administered. The best any author can do is transparently report the information in the paper, as we have done. We do not think this has adversely impacted the final article selection. |
| 1.2 | The quality of the studies ranged from 45%-96% by CCAT appraisal tool. Did the author consider excluding some poor-quality studies based on the evaluation? | In reality only one paper could be classed as poor quality (Cohen-Kettenis et al 2002), and this was taken into account in narrative reporting of results. In relation to quantitative analysis, Table 3 shows this paper was excluded from pooled analysis of age data due to overlap with other samples. |
| 1.3 | Since some of the goals cannot be met given the current evidences/literatures, I recommend the authors delete some unmet goals such as prevalence of GD and age of onset, and adjust the content accordingly. It is not adequate to just discuss it without having enough evidences handy. A follow-up question is whether it’s ready to review GD so thoroughly or should the manuscript be simplified to a narrowed scope? | We respectfully disagree with the reviewer’s suggestion. It is important that a systematic review set its questions *a priori* without any prejudice as to what we might expect to find. We consider it a key finding of this review that it is not possible to answer certain questions based on the quality / nature of the existing literature. We are pleased to see this view is supported by the other two reviewers, who support the need for this evidence gap to be highlighted. |
| 1.4 | Page 6, methods, study selection part. “Following initial full text screening, all remaining papers were assessed by a second reviewer to reduce the risk of inclusion bias.”. How could the authors be certain for the full inclusion of existing literatures when there was no independent search from at least two authors? | We are confident that the initial full text screening was robust as we were working to a very clear set of criteria where the eligibility was quickly determined by checking one or two details within the papers (e.g., the paper included participants up to age 45 with no differentiated data for adolescents). The need to have a second reviewer at the final screen was due to the eligibility being more difficult to determine at this later stage (i.e., the paper included only a mean and SD for age, making top age range more difficult to confirm). |
| 1.5 | Page 6, methods, study selection part. It was mentioned the overlaps were assessed, did they fully or partially overlapped? Please provide more details of the evaluation and extent of overlapping if possible. | Figure 2 provides a full summary of the nature and extent of study overlap in as far as we are able to report. We can only estimate the extent of true overlap based on dates and locations of samples, as papers rarely reported where known overlap occurred (see p7, second paragraph under ‘Results’). So although we agree with the reviewer that more data would have been useful, it was the quality of the literature rather than our methods which prevented us reporting further. |
| 1.6 | One page 9, paragraph 2. How could the age of onset range from 1 to 15 year old? I doubt if someone can be diagnosed with GD at the age of 1. | We do not disagree with this view. We were objectively reporting what the included papers chose to report. Our analysis showed that the data on age of onset was poor and that we were not able to adequately answer that question, so this will not have adversely impacted the review outcome. |
| 1.7 | Page 10, paragraph 3. How did the author deal with the unclear definition of “referral”? what about the referral could vary in different countries? | We agree with the author that this is problematic and have transparently reported this difficulty in the manuscript (p10, paragraph 3). This is again an issue with the quality of the literature rather than our methods. |
| 1.8 | In terms of the searching strategies. In Table 1. Could the authors explain the different search terms applied to different databases? Why didn’t the authors do a hand search? How did the authors pick databases to search? There are also some major archives such as PubMed and Scopus that weren’t included. | The search terms needed to be slightly altered for each database due to the way the database algorithms are configured. This is common practice in a systematic review. We selected databases and tailored searches based on the advice of our subject librarian. There is no need to include all available databases when overlap is so considerable. PubMed is the free interface to Medline, so there would be nothing gained by including it separately. Medline and Embase are both included within Scopus, although Embase has greater coverage. Thus we have adequately covered the available literature with this choice of databases. We did not conduct a full hand search of included texts as the volume of literature was so great. However, we checked whether any relevant texts cited in our review of papers had been included in our sample, and they had (either excluded at an earlier stage, or included in the final sample). |
| 1.9 | figure 1. please fill in the number for right upper frame (additional records…). So as fixing the records excluded #1 and specifying each items under full-text articles excluded. | Thank you for noticing this oversight. We have corrected the Figure. |
| 1.10 | In figure 2, questions 3a has zero literature, is it worth keeping it? | Please see our response to comment 1.3. |
|  |  |  |
| 2.1 | Given the scant amount of good actionable data I would prefer to see this paper as a call to action and perhaps even a series of specific proposals on how one should go about addressing the question of GD prevalence in the adolescent populations. | Thank you for this positive comment. We prefer to keep the paper as a systematic review given that it objectively demonstrates what the extant literature can or cannot say with regard to GD in adolescents. We have made recommendations in the Discussion section regarding the direction future research should take. |
|  |  |  |
| 3.1 | Abstract. The authors employ the term “natal gender.” It might be more parsimonious to consistently refer to natal sex (male/female), which is both immutable and for all intents and purposes, binary (despite low frequency of DSD in the population). The use of the term “natal gender” occurs again on page 6, for example, when describing what information was recorded from reports included in the review, as well as in Table 2, and on page 9 (Discussion). Similarly, the term “natal gender ratio” is used on page 8 (although I suspect this terminology is from the paper cited, being specific about a natal sex ratio might be advisable). | Thank you for this useful observation. We have changed these references to ‘natal sex’. |
| 3.2 | Introduction. A bit more terminological consistency would great aid readability of the entire manuscript. For example, the authors state, “There has been a parallel increase in demand for interventions to transition from one gender to another, particularly from female to male.” Here, sex and gender terms seem to be somewhat mixed. An individual cannot change their sex (chromosomes, genitalia, or likely most relevant, their gametes), although we can make social decisions to recognize that not all male individuals are men, and not all female individuals are women. As the authors note in the next paragraph, sex and gender are not synonymous. This differentiation may be a good place to introduce terminological consistency, wherein male/female always refer to sex, whereas boy/girl and man/woman are only used to refer to gender. I understand that this may deviate from the typical use in many contexts (legal, medical, etc.), but I think the more that such differentiated terminology is employed, the more it (hopefully) diffuses into dialogues about these subjects. Last, as a sidenote, I especially appreciate the author using the term “sex documented at birth” for the simple reason that medical professionals are rarely wrong about observing/documenting the biological sex of a newborn. Similarly, I am glad to see the authors use the terms “natal female” and “natal male.” These terms are accurate, precise, and indeed less cumbersome than many proposed alternatives. | Thank you for this useful observation. We have edited accordingly.  We are pleased the reviewer agrees with our rationale regarding the terms ‘sex documented at birth’ and ‘natal fe/male’ – these were given a lot of consideration. |
| 3.3 | Introduction. The authors use the term “imbalance” between sex and gender for some individuals. Discordance may be a more appropriate terminological choice. | We agree this is a useful suggestion and have edited accordingly. |
| 3.4 | The authors note a lack of systematic reviews regarding the epidemiology of GD. That said, I think it is important to acknowledge that some attempts at such reviews (e.g., Zucker, 2017; <https://www.publish.csiro.au/sh/sh17067>) have been undertaken, and do give some indication of both prevalence, and the trends that the authors are commenting on here. | We have amended the text to acknowledge previous relevant review work. |
| 3.5 | Characterizing the current discussion surrounding GD (and related issues) as an “intense international debate” is perhaps one of the greatest understatements I’ve ever seen in a manuscript. I want to commend the authors on their brave efforts to confront this topic head-on, and bring important data to bear on these discussions. | Thank you! |
| 3.6 | Regarding data from the Netherlands, so far as I know the Amsterdam clinic is the only gender identity service in the Netherlands. This means that the majority of adolescent GD cases in the Netherlands are highly likely to come into contact with the Amsterdam clinic. Obviously, some adolescents with GD may seek alternative services, but at the very least a lower boundary for the incidence/prevalence of GD can be estimated based on the number of adolescents seen by the Amsterdam clinic, as a proportion of all adolescents of that birth year (Statistics Netherlands—https://www.cbs.nl/en-gb—keeps fabulous, population level statistics on broad demographics within the population going back to roughly the 1950s). A similar argument might apply to the London clinic, although the UK has a considerably larger population than the Netherlands, making such estimates unwieldy and inaccurate in the absence of population level data, or valid random sampling. When discussing the prevalence of adolescent GD, the authors simply state that a precise estimate cannot be offered because population samples are not available. Surely some estimate can be given, especially in light of the authors speculating that there may be an increase in adolescents presenting for treatment. | Thank you – this is an important point. We have amended the text on p8 in Results and p10 in Discussion to acknowledge the likelihood of near population level data in these regions. We chose not to calculate likely prevalence / incidence based on these samples as they were reported to not be representative of the full population assessed at the clinics (the best source to use being de Graaf et al (2018) where data were available for 91.6% of the Dutch sample and 70.9% of the UK sample). |
| 3.7 | The authors mention a supplementary table detailing characteristics of excluded studies, but this supplement does not appear to be included in the reviewer-accessible submission files. This is important, as the authors state directly (page 10) that population-based survey data were excluded from the present sample. | Apologies – this table was omitted from the original submission. We have uploaded it with the revised manuscript. |
| 3.8 | I want to commend the authors on their attention to detail, and scientific caution when making conclusions based on the available evidence. Not all scholars studying GD are so careful. | Thank you – we very much appreciate the positive and constructive feedback. |

We look forward to hearing from you.

Yours sincerely

Lucy Thompson (on behalf of the authors)
