# Supplementary material for: A PRISMA systematic review of adolescent gender dysphoria literature: 1) Epidemiology
Source: PLOS Glob Public Health. 2022 Mar 9;2(3):e0000245. doi: 10.1371/journal.pgph.0000245 (PMC10021877; doi:10.1371/journal.pgph.0000245)
Supplement: Attachment — Submitted filename: response to reviewers.docx [file pgph.0000245.s003.docx]

**THE SAHLGRENSKA ACADEMY**

**Institute of Neuroscience and Physiology**

**Gillberg Neuropsychiatry Centre**

**Kungsgatan 12**

**411 19 Göteborg**

Dear Runsen Chen

We very much appreciate the further constructive comments from reviewers on our manuscript. We have addressed each of their points in turn in the table below. All documents have been uploaded in the appropriate format. We hope this will allow our paper to be published in the near future.

| 1.0 | I appreciate the authors' great efforts in reporting such valuable topics. There is scarcity of review in GD. But I have a few concerns regarding the revised manuscript. |  |
| --- | --- | --- |
| 1.1 | 1) handsearch was not conducted in this review, though it may have little effect on the final results, it still affect the stringency. | We appreciate that this point should be addressed within limitations of the methods. We have added a line in the Discussion section. |
| 1.2 | 2) this review included some obvious problematic data (eg. GD at the age of 1), I would recommend further confirmation with authors of original studies or remove them, which will theoretically impact the results. | The Matthews et al paper included a lower age of 1 year for age of onset, and 3 years for age of referral. Other papers (Heard et al, Holt et al) also included relatively young (4.7 and 5 years respectively) lower ranges for age of referral. In all cases, the mean ages were in line with other papers reporting narrower age ranges, suggesting these very young children could be considered outliers with data not impacting on overall outcomes. We therefore do not see value in removing them as part of the aim of this review is to transparently report the state of the current literature (see below). We have added a line in the Discussion to acknowledge this as a potential limitation. |
| 1.3 | 3) given the mixed quality of current studies included, the necessity of a systematic review should be revisited. It would be great to see if there are any new updates. | We respectfully disagree that a systematic review should only be conducted where the literature is known to be of an established quality or homogeneous standard. This knowledge of the literature quality can only be gained by conducting a systematic review, and so the heterogeneity in quality of the literature is a finding in and of itself. Further research now has a firmer basis on which to ensure better methodological / reporting quality. |
| 2 | I am pleased with the revised version. Thank you. | Thank you. |
| 3.0 | My overall impression of PGPH-D-21-00213_R1 remains positive, although the authors will also note that I have some remaining questions/considerations that I would have included in my initial evaluation if I had access to Table 6 upon first review. My largest concerns are about studies that narrowly missed the cutoff criteria. As I note below, I raise this largely because I suspect that many informed readers may have similar concerns. I hope that the authors will receive these comments in the way that they are intended—from a supportive colleague who nonetheless feels compelled to play devil’s advocate to help ensure the manuscript is as strong as possible given the importance of the topic. I want to thank the authors once again for their attention to detail, and the huge volume of work/documentation that this manuscript represents. | Many thanks for your thoughtful and kindly-expressed review. It is extremely helpful that you understand and appreciate the effort that has gone into this work, as well as the significant potential for negative feedback. Your attention to detail and devil’s advocate stance is exactly the tone needed and has been enormously welcome.  It is frustrating to have to exclude some good quality papers due to the need to apply some sort of age cut-off (where some papers will inevitably be excluded no matter what cut-off we chose). We hope that readers will appreciate there was no perfect way to capture all the relevant literature without bias and that we have tabulated all the ‘near-miss’ paper so the findings are as strong as they can be. |
| 3.1 | There are still some instances of a mismatch between using sex (male/female) as opposed to gender (man/woman/other) terminology. For example, the third sentence of the introduction states: “There has been a parallel increase in demand for interventions to transition from one sex to another, particularly from female to male.” Here seems like a place where “gender” might actually be the more appropriate term, because individuals are transitioning their gender with attempts to modify certain aspects associated with their biological sex (which cannot be modified). I know that exact wording in these kinds of manuscripts can be tricky—I’ve grappled with best approaches in my own work for years—so please bear with me. One potential solution is to slightly rephrase the sentence to read: “There has been a parallel increase in demand for gender transition interventions, particularly among natal females.” This highlights the sex documented at birth, without (unintentionally) suggesting that biological sex is an alterable characteristic. | Thank you for highlighting this. We have amended this sentence accordingly and checked the manuscript for other such instances. |
| 3.2 | Related to my point above, the second paragraph of the introduction states, “For most people who do not have abnormalities of the external genitalia, sex is documented at birth and, in the majority population, this is consistent throughout life.” This seems to give the impression that DSDs might be under discussion, where individual’s genital morphology conflicts with their chromosomal sex, and ultimately their adult gender identity. I would contend that, excepting DSDs, an individual’s sex is always consistent throughout their life because it is an immutable characteristic that cannot change even if a gender transition is undertaken. One solution would be to move this sentence to the end of the subsequent paragraph, and slightly rephrase it to read: “For most people who do not have abnormalities of the external genitalia, sex is documented at birth and, in the majority population, sex and gender are consistent throughout life.” | Thank you for this thoughtful suggestion. We have amended accordingly. |
| 3.3 | Some papers which were excluded stand out as likely candidates for inclusion. Indeed, I think Table 6 is most likely to be critiqued by other experts in the field, with quite a few exclusion decisions questioned. Although I appreciate a cautious, rigorous approach, I worry that some exclusions have been overly rigid. For example, the authors exclude Aiken et al. (2015), noting in Table 6 that it was unclear if GD was clinically verified. This is not true. For the Canadian sample in that paper, the authors note, “Depending on the year of assessment, DSM-III, DSM-III-R, or DSM-IV, criteria were used to diagnose Gender Identity, Disorder (GID) or Gender Identity Disorder Not Otherwise Specified (GIDNOS)” (Aiken et al., 2015, p. 757). This seems like a clear indication of assessment and criteria to me, at least for the Canadian data. Similarly, the Dutch sample notes an “age at the time of assessment” (p. 760). Given that these assessments occurred at a specialist GD clinic, and the sample is adolescents with GD, it is unclear what else could be meant aside from a clinical assessment of GD. Although the eldest participant in these data was 19, the vast majority of participants were evaluated in adolescence, as attested by Aiken et al.’s (2015) title, abstract, and methodology. Achille et al. (2020) was excluded because the mean age was 16.2±2.2, which seems to indicate ages of 18.4 were present (i.e., mean + sd). It is worth pointing out that a standard deviation such as this is possible, even if no individual is over 18. For example, the series of “ages” 17, 17, 17, 17, 11 has a mean of 15.8 and a SD = 2.4, even though no individual is actually over 18. Relatedly, excluding Alberse, et al. (2019) because the “max age 18.03” strikes me as overly restrictive. I understand that the authors have chosen a “hard line” of 18 as an upper-bound for age cutoffs, but excluding studies that are clearly relevant because the maximum reported age of a participant is above 18 seems to cut against the spirit of why age cutoffs are used in the first place, particularly when the authors themselves acknowledge that there is often a delay between initial assessment and treatment/intervention. I worry that there is a lot of baby being thrown out with this bathwater. Age cutoffs are necessarily arbitrary, and excluding relevant data that largely speak to adolescent GD, but include some individuals who may have reached age of majority (depending on jurisdiction) seems overly restrictive. I’m not entirely convinced that excluding an entire sample that is clearly composed predominantly of adolescents, simply because one or two individuals are 18, is the best course of action. As another example, studies like Becker et al. (2018) were excluded because “No separate data on adolescents” and the study includes those who are adults. However, Becker et al. (2018) consistently report data separated by their adolescent and adult sample. Last, studies like Jarin et al., 2017, were once again excluded because some participants were > 18 years old. While true on a surface level, much of this data includes long-term follow-up of initial adolescent assessment, with some of these individuals being tracked into adulthood. Although such a study can’t inform prevalence estimates, the ratio of male and female adolescents, and rough year of data collection, can nonetheless inform sex ratios of adolescent GD across time. That said, many of these papers come from a handful of world locales, and much of their data would be represented in other publications, so if reasons for exclusion include overlap with other work published by certain research groups, that is justifiable and should be noted. Many of these research groups (e.g., the Canadian group, the Dutch group, etc.) have also published summary data on long-term trends in GD, which are arguably the best sources of data for the present investigation.  As I write this, I’m not even sure myself whether this concern is something that should necessarily be addressed by the authors. The authors have been transparent about their search strategies and inclusion criteria, and scholars who might use alternative approaches are welcome to undertake that work themselves. I simply raise this here because I strongly suspect these (and similar) points will be raised if the work is published in present form. | We appreciate this feedback and agree that the paper may attract negative critique to this effect. We had to take the decision to be consistent in application of our *a priori* criteria and had to exclude papers we think have made a useful contribution and would otherwise very much have liked to include. We have added some text to the Discussion section to acknowledge this more strongly, which may go some way to allaying criticism.  Re. Aitken et al (2015), we agree it was a shame to exclude this paper. To clarify, Sample 1 (Canadian) was excluded as the eldest was 19 years old. Sample 2 (Netherlands) was excluded as diagnostic status was unclear. We have made this distinction clearer in Table 6.  Re. Achille et al (2020) – although it may be possible that the sample did not include anyone over 18, it was similarly possible that it did. We judged that it was best to use a ‘balance of probabilities’ approach to assessing eligibility, and on balance in this case it was probable that there were patients over 18.  Re. Alberse et al (2019) – we agree it is a shame to exclude a paper where the maximum age was so close to our cut-off. We thought it important to be consistent, however, as other papers would have been excluded at an earlier stage of screening had they reported having participants aged over 18 in the Abstract, for example. Including papers at the final full text screen on the basis of their not quite meeting criteria but including interesting findings would have risked adding bias against papers excluded at an earlier stage.  Re. Becker et al (2018), you are right that adolescent and adult samples were treated separately. However, adolescence in the Becker paper was classed as up to age 21. So we are applying our criteria for adolescence rather than theirs in excluding them from our sample.  Re. Jarin et al (2017) – I would apply the same reasoning as with Alberse – whilst more flexible application of criteria may have allowed us to say more, there would have been a risk of bias in relation to studies excluded at an earlier stage of screening.  We have used these as examples of excluded papers that could have been included were it not for our strict criteria in the added section of the Discussion. We do not wish to give the impression that the importance of these papers has not been considered. There are several non-systematic reviews available that will include these high-profile papers. Our objective was to take in the totality of the literature, and to examine the state of the evidence once strict criteria were applied. We expect others will not agree with this approach, but our point is to make a call for improved quality of reporting in this literature, as crucial clinical policy relies on accurate reporting in the interests of distressed young people.  Thanks again for your positive reflection on these points. I hope that in addressing these with added text in the paper, we have made our case more clear for the need to improve quality of reporting to allow reviews to be more precise in synthesising the evidence. |
| 3.4 | There is a noticeable lack of reference to other scholarship noting shifts in the sex ratio of GD. For example, Zucker and Aiken (2020; <https://iris.unito.it/retrieve/handle/2318/1711667/532720/Boof-of-abstracts-EPATH2019.pdf#page=69>) or Steensma et al. (2018; <https://www.tandfonline.com/doi/abs/10.1080/0092623X.2018.1437580?journalCode=usmt20>). Both of these studies are consistent with the authors’ assertion that a change in sex ratio for adolescent GD has occurred over the past several decades. I think these inclusions are important, because converging lines of evidence aid in shoring up any scientific conclusion. | Thank you for this. We have included reference to the Zucker et al paper as an indication of recently emerging evidence that did not meet our criteria, and the Steensma et al paper as an indication that the shift in sex ratio applies to children as well as adolescents. |

We look forward to hearing from you.

Yours sincerely

Lucy Thompson (on behalf of the authors)
